# Comment on Miyamoto et al. Laryngopharyngeal Mucosal Injury Due to Nasogastric Tube Insertion during Cardiopulmonary Resuscitation: A Retrospective Cohort Study. *J. Clin. Med.* 2024, *13*, 261

**DOI:** 10.3390/jcm13123447

**Published:** 2024-06-13

**Authors:** Andrew Maxwell

**Affiliations:** Department of Anaesthesia, Intensive Care and Pain Medicine, Cork University Hospital, Wilton, T12 DC4A Cork, Ireland; andrew.maxwell@hse.ie

I read with great interest the study from Miyamoto et al. [1]. The authors are to be commended for their collection of high-quality data, images and videos while performing procedures under challenging circumstances and in time-critical scenarios. This study provides valuable new insights into the insertion of nasogastric tubes (NGTs) in intubated patients, particularly regarding the sharp points formed by kinked NGTs and their correlation with mucosal injury. However, two points for potential debate are outlined below.

Firstly, the authors concluded that in cases where smooth insertion of an NGT has not been successful in under 1 min, the attempt should be postponed, and another technique used instead. They further state that “whenever blind NGT insertion is difficult, we should switch to other methods, without any unnecessary persistence”. This conclusion seems logical at first glance, and is based on the fact that severity of mucosal injury was correlated with longer time to insertion. However, an important finding in this study was that one NGT insertion in the “smooth” group (1 of 67, or 1.5%) resulted in misplacement in the respiratory tract. This misplacement rate is in keeping with other studies of NGT insertion, although on the lower end of the range, typically 1.5–3.2% regardless of the type of NGT used [2,3,4,5]. The precise time taken to insert the NGT that was noted to be misplaced in the trachea was not reported, but obviously did not meet the predetermined cutoff of 2 min to be classed as “difficult”. Additionally, in three cases in the VL-converted group, the NGT was seen to be advancing towards the trachea during insertion. It is important to consider the possibility that these patients may also have experienced intrapulmonary misplacement during the 2 min duration blind insertion attempts.

When NGTs are misplaced into the respiratory tract, patients are at high risk of iatrogenic morbidity and mortality [6]. Complications may include pneumothorax in up to 40% of cases [3], pneumonia, lung abscess, and others [7,8]. It is therefore clear from the data presented in this study, consistent with previous literature, that blind insertion of NGTs will sometimes result in intrapulmonary misplacement. Additionally, easy or timely insertion to the desired length does not necessarily mean that safe or correct placement has been achieved, and difficult insertion is not the only instance where harm can occur from the procedure.

The second interesting finding from this study is that in the 17 patients in whom insertion was deemed to be difficult or not possible within 2 min, video laryngoscopy (VL) facilitated successful NGT insertion into the oesophageal inlet in all 17 patients, avoiding the respiratory tract. In four of these patients, the NGT did not advance due to possible anatomical variations in the lower regions of the oesophagus, but the NGT was nonetheless correctly directed toward the oesophagus using the real time VL images and appropriate manoeuvres. The time taken for VL-assisted insertions after converting was similar to blind insertions (54.8 ± 3 s vs. 48.8 ± 4 s, respectively), which in real life terms is a negligible difference.

The VL technique therefore provided several distinct advantages, while taking a similar time to successful insertion. Firstly, the real-time VL images allowed correction of potential tracheal misplacement before it occurred in three cases. Secondly, it allowed the identification of the NGT kinking and forming sharp points, which was observed to contribute to mucosal injury in this study. This information can be used to prevent mucosal injury and bleeding caused by NGT kinking as it is being observed during VL use, in contrast to blind insertion where it may not be appreciated. Thirdly, it allowed early identification of those NGTs which were impacting in the middle or lower oesophagus during VL, which is useful in identifying patients in which NGT insertion may not be possible at all using standard techniques. This may potentially save time and effort relating to futile confirmatory tests (such as chest X-rays) when correct placement has been deemed unlikely based on VL.

The authors’ conclusion that VL-assisted insertion should be used when blind insertion proves to be prolonged or difficult is logical based on the results from this study. However, in the overall context of NGT insertion in all intubated patients, it may inadvertently reinforce the misconception that blind insertion is a safe initial strategy. Instead, an alternative conclusion from this study may therefore be that VL-assisted NGT insertion appears to be safer and more accurate than blind insertion in intubated patients during CPR, while taking a similar amount of time to perform, and consequently blind insertion should not be recommended as a safe insertion technique. Currently, there are no clinical guidelines which recommend one insertion technique over another, and clinicians responsible for NGT insertion in intubated patients, including during CPR, must consider all available options. Studies like this one may help to form the basis for guidelines in the future. Laryngoscopy for intubation is a basic skill required for anaesthesiologists, critical care and emergency medicine physicians, and the skills are easily transferable to VL use for NGT insertion without significant additional training. Any specific training could be performed in airway mannequins or other simulated settings, which may help to further develop the skills required for VL-assisted NGT insertion before the transition to real clinical scenarios.

There are also disadvantages to VL use for NGT insertion, however, such as haemodynamic changes or increased intracranial pressure, and the risk of dental injury. As the patients in this study were in cardiac arrest and receiving CPR, haemodynamic variations were not applicable in these cases. However, rates of dental injury from laryngoscopy were not reported by the authors, and it is possible from the small sample size (*n* = 17) and experience of the physicians performing the procedure that no dental injury occurred. Video laryngoscopy is also not universally available or ubiquitous due to the cost of the devices, and its availability may influence the choice of technique. Finally, in some patients requiring NGT insertion, VL may not be possible due to restricted mouth opening or variations of facial or airway anatomy, or prone positioning in the ICU. Blind insertion may be justified in certain circumstances such as these; however, other potential methods and solutions should still be explored.

## References

[1] Miyamoto K., Takayasu H., Katsuki S., Maeda A., Suzuki K., Nakamura M., Hida N., Sambe T., Yagi M., Sasaki J. (2024). Laryngopharyngeal Mucosal Injury Due to Nasogastric Tube Insertion during Cardiopulmonary Resuscitation: A Retrospective Cohort Study. J. Clin. Med..

[2] Taylor S., Manara A.R. (2021). X-ray Checks of NG Tube Position: A Case for Guided Tube Placement. Br. J. Radiol..

[3] de Aguilar-Nascimento J.E., Kudsk K.A. (2007). Clinical Costs of Feeding Tube Placement. JPEN J. Parenter. Enter. Nutr..

[4] Rassias A.J., Ball P.A., Corwin H.L. (1998). A Prospective Study of Tracheopulmonary Complications Associated with the Placement of Narrow-Bore Enteral Feeding Tubes. Crit. Care.

[5] Sorokin R., Gottlieb J.E. (2006). Enhancing Patient Safety During Feeding-Tube Insertion: A Review of More than 2000 Insertions. JPEN J. Parenter. Enter. Nutr..

[6] Sparks D.A., Chase D.M., Coughlin L.M., Perry E. (2011). Pulmonary Complications of 9931 Narrow-Bore Nasoenteric Tubes during Blind Placement. JPEN J. Parenter. Enter. Nutr..

[7] Long M., Machan M., Tollinche L. (2017). Intraoperative Gastric Tube Intubation: A Summary of Case Studies and Review of the Literature. Open J. Anesthesiol..

[8] Halloran O., Grecu B., Sinha A. (2011). Methods and Complications of Nasoenteral Intubation. J. Parenter. Enter. Nutr..

